# Possible Male Reproduction Complications after Coronavirus Pandemic

**DOI:** 10.22074/cellj.2021.7982

**Published:** 2021-08-29

**Authors:** Maryam Hezavehei, Bahare Shokoohian, Mohammad Hossein Nasr-Esfahani, Anastasia Shpichka, Peter Timashev, Abdolhossein Shahverdi, Massoud Vosough

**Affiliations:** 1. Department of Embryology, Reproductive Biomedicine Research Center, Royan Institute for Reproductive Biomedicine, ACECR, Tehran, Iran; 2. Department of Regenerative Medicine, Cell Science Research Center, Royan Institute for Stem Cell Biology and Technology, ACECR, Tehran, Iran; 3. Department of Reproductive Biotechnology, Reproductive Biomedicine Research Center, Royan Institute for Biotechnology, ACECR, Isfahan, Iran; 4. Institute for Regenerative Medicine, Sechenov University, Moscow, Russia

**Keywords:** COVID-19, Fertility, Male Reproduction, SARS-CoV-2, Semen

## Abstract

Coronavirus disease 2019 (COVID-19), as a severe respiratory disease, affects various tissues and organs. The specific SARS-CoV-2 receptor, angiotensin-converting enzyme 2 (ACE2), is highly expressed in male gonads. Thus, male reproductive tissues could be a potential target for virus colonization. We performed a comprehensive search in PubMed and Google Scholar to retrieve relevant articles published till 15 April 2021. The keywords used were: male fertility, male reproductive health, semen parameters, sex hormones, SARS-CoV-2, and COVID-19. Validated evidence about the adverse effects of the SARS-CoV-2 infection on the male reproductive system is limited and few studies have reported semen analysis results or presence of viral RNA in semen samples of infected men. Nevertheless, alterations in reproductive hormones such as decreased level of testosterone (T) with raised luteinizing hormone (LH) have been reported in some patients. Although the impact of SARS-CoV-2 infection on the male reproduction health remains unclear, evidence suggests that male gonads may be potentially vulnerable to SARS-CoV-2 infection. In this article, we discussed the possible impacts of COVID-19 on male gonads, sex hormones, and semen quality and suggested preventive solutions.

## Introduction

The coronavirus disease 2019 (COVID-19) pandemic has led to a public health crisis affecting the world population (1). Coronaviruses are a group of human pathogenic viruses originated from animals. Severe acute respiratory syndrome coronavirus 2 (SARS-CoV-2) infection symptoms are fever, cough, shortness of breath and/or gastrointestinal disorders. While almost 80% of SARS-CoV-2 infections are mild, near 5 % are critical and death may occur in up to 3%. Older age and underlying disorders are major risk factors for severe SARS-CoV-2 infection (2). In addition to the abovementioned disorders, COVID-19 epidemic can also affect reproductive health (3). 

SARS-CoV-2 enters the host cells through the cellular receptors including angiotensin-converting enzyme 2 (ACE2), and the trans-membrane serine protease (TMPRSS) (4). Wang and colleagues reported that ACE2 is expressed in spermatogonia, Leydig and Sertoli cells, while TMPRSS2 is expressed more in Sertoli cells compared to spermatogonia cells (5). Also, Hikmet et al. (6) reported high expression levels of ACE2 in Leydig and seminiferous ducts cells. Expression of other coronavirus-associated receptors including basigin (BSG), alanyl aminopeptidase (ANPEP) and dipeptidyl peptidase 4 (DPP4) in spermatogonia stem cell (SSC) and co-expression of ANPEP and DPP4 in prostate gland have been reported recently (7). These findings suggested that male gonads could be vulnerable following SARS-CoV-2 infection which might consequently result in spermatogenesis impairment. Previous studies reported that certain viruses such as human immunodeficiency virus (HIV), hepatitis B and C viruses, papilloma, mumps, and SARS coronavirus (SARS-CoV) cause orchitis and leading to germ cell destruction and sterility (8, 9). However, there are few clinical reports on the association of SARS-CoV-2 infection and orchitis. In addition, studies suggested that coronavirus might have indirect effects on testicular function through immunological and inflammatory responses disturbing the hypothalamic-pituitary-testis axis (10). Understanding whether SARS-CoV-2 can infect testicular tissue, is important for assessing its possible adverse effects on male fertility. Therefore, the goal of this review is to discuss probable adverse impact of COVID-19 on male reproduction and show the possibility of fertility complications. Accordingly, we searched for the following keywords in Google Scholar and PubMed: COVID-19, SARS-CoV-2, or severe acute respiratory syndrome coronavirus 2 AND male fertility, testes, semen, testosterone (T), assisted reproductive technology (ART), or reproductive system. 

### Cellular and molecular aspects of COVID-19 effects in the testis

ACE2 is expressed in various human organs such as kidneys and gastrointestinal tract (11). In addition, ACE2 is highly expressed in the testis (2), particularly on Leydig cells. SARS-CoV-2 could effect on the renin angiotensin system (RAS) via ACE2. The RAS controls cascade of hormones and receptors in the multiple organ systems and balances blood pressure. The pathological processes in COVID-19 leading to overreaction of the renin-angiotensin-aldosterone along with clinical abnormalities such as adverse cardiovascular and respiratory in infected patients (12). Accordingly, this could be important for the pathogenesis effects of SARS-CoV-2 in men patients. Studies confirmed high levels of *ACE2* transcripts in spermatogonia cells, Sertoli and Leydig cells and a lower expression of *ACE2* in late spermatocytes and spermatids (5). Shen et al. (13) showed that the positive rate of ACE2 expression in infertile men was higher than normal by single-cell RNA-sequencing. This indicates that men with reproductive disorder may be simply infected by this virus, however there isn’t any study which directly prove this subject. In addition, Pan and colleagues showed a correlation between ACE2 expression and severe impairment in spermatogenesis. In addition, it has been reported that 18% of infected patients had a scrotal discomfort (14). Moreover, several studies have demonstrated the effects of SARS-CoV-2 infection on the testis including orchitis with fibrin micro-thrombi (15), mild lymphocytic infiltration, decreased the percentage of Leydig cells, and seminiferous tubular damage (16, 17). Therefore, high expression of ACE2 in the testis could facilitate viral entry and colonization, which may have negative impact on male fertility and possible reproductive impairments (18). *TMPRSS2* is strongly expressed in both early and late SSCs as well as Sertoli cells (7). *TMPRSS2* is also highly expressed in the prostate, seminal vesicles and epididymis, where its aberrant expression is associated with tumorigenesis (14). Singh et al. (7) experimentally showed that other receptors such as ANPEP (SARS-CoV), DPP4 (MERS-CoV) and BGS (an alternate receptor for both SARS-CoV and SARS-CoV-2) facilitate SARS-CoV-2 entry. Also, the highest transcript levels of coronavirus-associated receptors factors )SCARFs( have been showed in SSC and SPG. Taken together, these observations indicated that spermatogonial cells may be highly prone to SARS-CoV-2 infection.

### SARS-CoV-2 infection and male gonad function

Several studies reported low levels of T but high levels of luteinizing hormone (LH) and follicle-stimulating hormone (FSH) in male patients who infected with different viruses. In fact, viral infections could disrupt normal function in Leydig cells and decrease T to LH ratio (T/LH). These studies suggested that viruses might have an indirect impact on testicular function via modulation of the hypothalamic-pituitary-testis axis (19). 

Recently, the effect of SARS-CoV-2 infection was evaluated on gonadal function and male sex hormones; based on the results, LH level in serum was increased and ratios of FSH/LH and T/LH were decreased in recovered patients compared to the healthy men. However, other studies showed lower levels of total T and dihydrotestosterone in the patients with severe COVID-19 (20). Rastrelli et al. (20) reported that inflammatory cytokine storm in SARS-CoV-2 infection may be a potential risk factor for Leydig cells and would result in lower T levels, subsequently. Increased LH and decreased T/LH ratio may have occurred due to damage to Leydig cells and dysfunction of steroidogenic pathways after viral infection (21).

It seems that leukocytes and immune cells can pass the blood-testis barrier (BTB) and produce interferons that inhibit steroidogenesis and T production in the infected testis. Importantly, all above-mentioned symptoms are clinical phenotypes of primary hypogonadism. This phenomenon increases the risk of male infertility (22). 

Epidemiological data showed higher COVID-19 mortality rate in men compared to women, which may suggest an important role for T in determining gender-disparity in SARS-CoV-2 infection (3, 23). Previous studies showed that certain moods have adverse effects on sperm quality parameters such as motility, concentration and DNA integrity (23, 24).

Accordingly, the outbreak of COVID-19 can be associated with depression and posttraumatic stress disorder (PTSD), which lead to activation of the central stress response system via the hypothalamic-pituitary-adrenal (HPA) axis (25). Continuous stimulation of the HPA axis by stressors might lead to dysregulation of this axis. Because of the dynamic interactions between the HPA axis and the hypothalamic-pituitary-gonadal (HPG) axis, any alteration in this regard could induce abnormalities in the reproductive health (26). Wdowiak et al. (27) showed that depression and anxiety are associated with decreased level of sex hormone-binding globulin (SHBG), but increased secretion of cortisol and prolactin in sub-fertile males. 

It has been reported that SARS-CoV-2 infection can interfere with the production of FSH, T, and LH. Accordingly, serum LH level was higher in the COVID-19 patients and also the ratios of T: LH and FSH: LH were decreased in them compared to the control group (28). Ruan et al. (29) demonstrated that semen quality declined, while hormonal profiles remained normal in SARS-CoV-2 patients. Temiz et al. (30) evaluated sex hormone levels between COVID-19 patients before and after treatment and control group. Patients before treatment had significantly lower serum FSH, LH and T levels compared to the controls, which it could be consistent with the patient stress due to COVID-19.

### SARS-CoV-2 infection and semen quality

Until now, only Li et al. (31) reported SARS-CoV-2 RNA detection in 15.8% of semen samples from COVID-19 patients after recovery. Moreover, Pan et al. (14) reported 19% of patients had scrotal discomfort at the time of COVID-19 diagnosis. Holtmann et al. (32) showed that in COVID-19 patients with moderate symptoms, sperm quality decreased. In addition, Ma and colleagues evaluated the sex-hormones and semen characteristics in 12 infected men. They didn’t find any SARS-CoV-2 in semen samples. Although they observed a higher serum LH and a lower ratio of T to LH in the COVID-19 patients compared to the control group. However, 8 out of 12 patients had normal semen quality (28). Temiz et al. (30) also evaluated the effects of SARS-CoV-2 infection on semen parameters and serum male sexual hormones. They reported that SARS-CoV-2 was not detected in the semen samples of infected patients and also all the semen parameters and hormone levels were normal except sperm morphology in some patients compared to the control. However, the levels of the median serum FSH, LH and T showed significant decreased in the COVID-19 patients. It seems that SARS-CoV-2 can increase apoptosis rate in spermatogonia cells following increased oxidative stress and reactive oxygen species (ROS) production which lead to reduced semen quality. Furthermore, Gacci et al. (33) showed that despite the absence of viral RNA in the semen, 25% of the 43 infected men were oligocrypto-azoospermic, whereas8 were azoospermic and 3 were oligospermic after recovery from COVID-19. Moreover, 33 out of 43 patients (76.7%) showed pathological levels of IL-8 in their semen, although the semen quality of these men were unknown before infection. The previous studies in terms of the detection of the SARS-CoV-2 virus in semen showed conflicting results (Table 1), thus further studies are needed to understand sexual transmissibility of the SARS-CoV-2 and its impact on sperm quality (34).

### SARS-CoV-2 infection and inflammatory responses

Previous studies have reported that SARS-CoV-2 could damage the male reproductive system by inflammatory caused by cytokine storm (35). The potential mechanisms of COVID-19-induced immunopathology in terms of immune cells including higher levels of neutrophils, lower levels of eosinophils, basophils, and monocytes. Depletion and exhaustion of lymphocytesincreased production of certain cytokines including IL-1β, IL-6, and IL-10 are other possible mechanisms of COVID-19 induced immunopathology. 

**Table 1:** SARS-CoV-2 infection and semen quality


Patients (n)	Viral RNA in semen	Reproductive system symptoms	Semen quality	Reference

34	Negative	Scrotal discomfort (19%)	Not assessed	(14)
20	Negative	Not reported	Decreased sperm concentration and motility	(32)
38	Positive (15.8%)	Not reported	Not assessed	(25)
38	Positive (26.7% in the acute stage of infection), Positive (8.7% in recovery)	Not reported	Not assessed	(31)
12	Negative	Not reported	33.3% (low sperm motility with higher DFI)	(28)
74	Negative	Scrotal discomfort in one patient	Decreased semen quality	(29)
30	Negative	Not reported	Decreased sperm morphology	(30)
43	Negative	Not reported	5% of the men were oligo-crypto-azoospermic, 8 were azoospermic and 3 were oligospermic	(33)


SARS-CoV-2; Severe acute respiratory syndrome coronavirus 2 and DFI; DNA fragmentation index.

In addition, SARS-CoV-2 damages lymphatic organs, such as the spleen and lymph nodes, leading to lymphopenia (36). Based on the recent reports, inflammatory responses in SARS-CoV-2 infection could be classified as primary and secondary responses. Primary inflammatory responses happen after viral infection, prior to the advent of neutralizing antibodies (NAbs). Secondary inflammatory responses start with the generation of adaptive immunity and NAbs. In fact, the virus-NAb complex causes FcR-mediated inflammatory responses, skewing macrophage responses, cellular damages and acute lung injury (37). 

**Fig.1:**
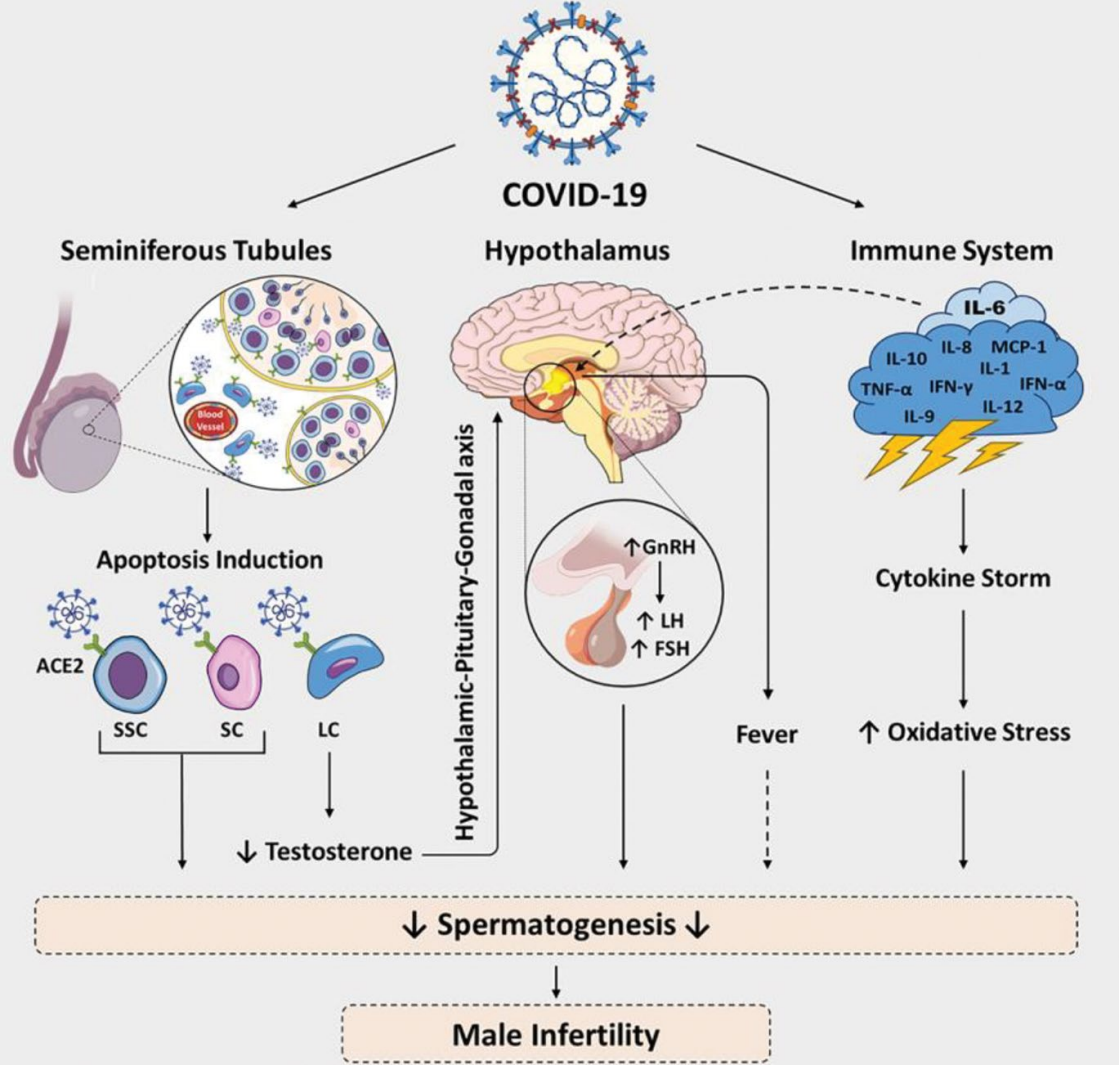
Risk factors, mechanisms and pathophysiology of COVID-19 on the male reproduction. ACE2; Angiotensin-converting enzyme 2, SSC; Spermatogonia stem cell, LC; Leydig cell, SC; Sertoli cell, GnRH; Gonadotrophin-releasing hormone, LH; Luteinizing hormone, FSH; Follicle-stimulating hormone, and IL; Interleukin.

The main cause of COVID-19 severity is the cytokine storm which is associated with increasing serum levels of interleukin-6 (IL-6), IL-7, IL-8 and tumor necrosis factor alpha (TNF-α) (38, 39). Systemic viral infections such as influenza can indirectly interfere with male reproduction. Their detrimental effects can be exerted by fever, immune cell activation, and elevated inflammatory mediators (cytokines and interferons), which may have inhibitory effects on spermatogenesis and steroidogenesis (Fig .1) (8).

Furthermore, increased IgG precipitation and leukocyte infiltration were shown in testicular interstitial tissue following viral infection (7). High levels of inflammatory cytokines produced by leukocytes, T cells and macrophages following viral infections, can deteriorate spermatogenesis and steroidogenesis (22). For other coronaviruses such as SARS-CoV, reproductive system damage was observed in males as well. Accordingly, Xu and colleagues showed IgG deposition in seminiferous epithelium and degenerated germ cells and Sertoli cells in SARS-CoV patients. However, they did not observe SARS-CoV related mRNA in the testicular tissue (8). The observed effect could be due to an immune-mediated response to SARS-CoV infection.

Moreover, the gene ontology (GO) analysis showed that genes related to immune reaction were increased in ACE2- positive Leydig and Sertoli cells, while reproductive-related genes were down-regulated (6). It was suggested that increased endothelial cell death and vascular permeability could promote production of pro-inflammatory cytokines, which can lead to dysfunction of the renin-angiotensin system (RAS), and further exacerbate the inflammation (40). In addition, inflammation caused by SARS-CoV-2 infection can increase immune cell recruitment in testicular tissue and affect the BTB integrity (22). Epididymitis was reported using color Doppler ultrasound in 42.3% of mild-to-moderate COVID-19 men, which 54.5% of them presenting with enlarged epididymis head, and 19.2% had bilateralism. It could be an indirect deleterious effect of inflammation that targeted the epididymis (41). Further studies are needed to provide enough evidence about the role of the inflammatory responses in male infertility under SARS-CoV-2 infection.

### COVID-19 and male fertility

Documented information about the adverse effect of SARS-CoV-2 infection on male fertility is limited. Similar to other coronaviruses, several mechanisms may be involved in testicular dysfunction and male infertility caused by SARS-CoV-2 infection. It was shown that some members of the coronavirus family can cause orchitis and testicular dysfunctions. Pathological findings from SARS-CoV-infected patients showed that this virus induces apoptosis in spermatogonia cells and, increases leukocyte infiltration (8). Influenza viruses promote oxidative stress (42) that negatively influences spermogram parameters including motility and viability (39). In this context, it can be assumed that SARS-CoV-2 may affect sperm function through systemic inflammation leading to enhanced oxidative stress. Only one study reported that SARS-CoV-2 can decrease sperm concentration and motion parameters in infected patients (43), while other studies recommended assessing the adverse effects of SARS-CoV-2 on semen quality in infected and recovering patients. Fever that associated with viral infections causes spermatogenesis impairment, e.g. SARS, leads to increased apoptosis in testicular germ cells (44) and disrupted spermatogenesis (45). The percentage of asymptomatic infection in the children under 10 years old is about 16% and negative effects of infection may be a serious challenge for their future reproductive health. Therefore, long-term side effects of high fever on male fertility should be considered following SARS-CoV-2 infection. SARS-CoV-2 may cause testicular dysfunction by two distinct effects, direct or indirect, due to the presence of receptors such as ACE2 in the testis or immunological and inflammatory responses and dysregulated male sex hormones. However, supportive data for direct adverse effects are controversial (Fig .1) (37). 

### The effect of COVID-19 treatment on male fertility

Currently, there are no highly effective therapeutic solutions to control SARS-CoV-2. However, antiviral drugs such as interferons, ribavirin, lopinavir/ritonavir, antibiotics such as moxifloxacin and azithromycin, and glucocorticoids are recommended for COVID-19 treatment (2, 25) .Several studies noted that some of the mentioned drugs have adverse effects on male reproductive health (46). Almasry et al. (47) have indicated that ribavirin decreased T levels and impaired spermatogenesis in animal models. Additionally, sperm abnormalities and decreased sperm count were observed in rats treated with ribavirin (48, 49). Lopinavir/ritonavir could also impair spermatogenesis in male rats (50). Glucocorticoids could destroy cell junctions in spermatogenic epithelium and the BTB, which cause germ cell apoptosis in testicular tissue (46). Moreover, it was reported that excessive use of disinfectants could lead to a decrease in sperm count and quality in mice (51). Side effects of drugs used for the treatment of COVID-19 on male reproductive health should be considered cautiously. 

### Managing the possible effects of COVID-19 on male reproduction

Management of SARS-CoV-2 possible adverse effects on male fertility is complex due to the underlying pathophysiology and associated co-morbidities. It appears that SARS-CoV-2 may have indirect impacts on the male reproductive system by inducing inflammatory storm (25) and infected male fertility remains at risk due to the increased oxidative stress level. Therefore, immune response modulation and inflammation management were suggested for the disease management (52-54). Treatment with various antioxidants including vitamin E, C and D as well as selenium, and zinc, which positively modulate inflammation and oxidative stress, may be warranted (55- 57). In addition, it was reported that supplementation with omega-3 fatty acids is associated with better testicular function (58). Antioxidant treatment can reduce the risk of DNA damage in sperms and improve fertility through improving semen parameters (55, 56). Therefore, administration of antioxidants and immunosuppressants could be a novel therapeutic strategy against possible male subfertility induced by COVID-19. However, currently there is no approved guideline for nutrition in the male patients. In addition, because of global financial crisis during SARS-CoV-2 infection many fertile couples decide to postpone the time of parenthood. Therefore, it may cause primary or secondary infertility in the future.

Additionally, selective infecundity may cause biological infertility which might be linked to short and mid-term increase in global infertility (59). Generally, there are a few proposals for individualized clinical management in ART services to alleviate the adverse effect of the coronavirus pandemic. The global guidelines such as the American Society for Reproductive Medicine (ASRM) and the Society for Assisted Reproductive Technology (SART) recommended caution to couples planning natural pregnancy or assisted reproduction (60). ART treatment should be carried out for infertile patients who need immediate treatment. Anthological services such as fertility preservation should be available during any treatment. Patients and healthcare providers should use proper personal protective equipment. It seems training ART staff, following social distancing protocols, psychological support, and prioritizing of COVID-19 patients are necessary (61). In addition, because of global financial crisis during SARS-CoV-2 infection many fertile couples decide to postpone the time of parenthood. Therefore, it may cause primary or secondary infertility in the future. Additionally, selective infecundity may cause biological infertility which might be linked to short and mid-term increase in global infertility (59). 

## Conclusion

So far, there is limited evidence about SARS-CoV-2 pathogenicity on male reproductive health. ACE2 protein as a functional receptor for SARS-CoV-2 is highly expressed on testicular cells. TMPRSS2 receptor also has a high expression level in Sertoli cells. Thus, male gonads may be potentially vulnerable to SARS-CoV-2 infection. Although limited data is currently available, detection of SARS-CoV-2 virus in semen of some patients may indicates the possibility of sexual transmission in COVID-19. Follow-up studies are required to investigate this possibility. Moreover, DNA fragmentation and defective sperm function might be occurred during SARS-CoV-2 infection because of inflammation responses and increased oxidative stress in the patients. DNA damage in sperm can lead to a lower fertility rate or idiopathic infertility. On the other hand, recent reports revealed that all the parameters in the semen samples were similar between the COVID-19 patients and the healthy control group and the only significant reduction in the percentage of the sperms with normal morphology has been observed in the COVID-19 patients, which could be the result of fever in the infected men. In addition, epididymitis could be as an indirect effect of inflammation in COVID-19 men, thus semen analysis and testicular evaluation are recommended in the patients after recovery. Some antiviral drugs have adverse impact on the male reproductive health system. Hence, further studies should investigate in terms of a safe treatment strategy for male fertility following SARS-CoV-2 infection. Furthermore, psychological consultation and clinical information should be provided to couples during the outbreak of COVID-19 and contraception could be suggested for the patients who were COVID-19 positive and received antiviral treatment. Nonetheless, further research is needed for assessing the impact of SARS-CoV-2 infection on testicular tissue, sex hormones and sperm quality. 
